# Hearing screening for school children: utility of noise-cancelling headphones

**DOI:** 10.1186/1472-6815-13-6

**Published:** 2013-05-24

**Authors:** Ada Hiu Chong Lo, Bradley McPherson

**Affiliations:** 1 Division of Speech and Hearing Sciences, Faculty of Education, University of Hong Kong, Pokfulam Road, Pokfulam, Hong Kong

**Keywords:** Background noise, Headphones, Hearing loss, Hearing screening, School children

## Abstract

**Background:**

Excessive ambient noise in school settings is a major concern for school hearing screening as it typically masks pure tone test stimuli (particularly 500 Hz and below). This results in false positive findings and subsequent unnecessary follow-up. With advances in technology, noise-cancelling headphones have been developed that reduce low frequency noise by superimposing an anti-phase signal onto the primary noise. This research study examined the utility of noise-cancelling headphone technology in a school hearing screening environment.

**Methods:**

The present study compared the audiometric screening results obtained from two air-conduction transducers—Sennheiser PXC450 noise-cancelling circumaural headphones (NC headphones) and conventional TDH-39 supra-aural earphones. Pure-tone hearing screening results (500 Hz to 4000 Hz, at 30 dB HL and 25 dB HL) were obtained from 232 school children, aged 6 to 8 years, in four Hong Kong primary schools.

**Results:**

Screening outcomes revealed significant differences in referral rates between TDH-39 earphones and NC headphones for both 30 dB HL and 25 dB HL criteria, regardless of the inclusion or exclusion of 500 Hz results. The kappa observed agreement (OA) showed that at both screening intensities, the transducers’ referral agreement value for the 500 Hz inclusion group was smaller than for the 500 Hz exclusion group. Individual frequency analysis showed that the two transducers screened similarly at 1000 Hz and 2000 Hz at 25 dB HL, as well as at both 30 dB HL and 25 dB HL screening levels for 4000 Hz. Statistically significant differences were found for 500 Hz at 30 dB HL and at 25 dB HL, and for 1000 Hz and 2000 Hz at 30 dB HL. OA for individual frequencies showed weaker intra-frequency agreement between the two transducers at 500 Hz at both intensity criterion levels than at higher frequencies.

**Conclusions:**

NC headphones screening results differed from those obtained from TDH-39 earphones, with lower referral rates at 500 Hz, particularly at the 25 dB HL criterion level. Therefore, NC headphones may be able to operate at lower screening intensities and subsequently increase pure-tone screening test sensitivity, without compromising specificity. NC headphones show some promise as possible replacements for conventional earphones in school hearing screening programs.

## Background

There are two main types of audiometric screening that target children—newborn hearing screening and school hearing screening. Since between 1% and 14% of children have permanent or transient hearing loss, respectively, at school [1] and studies have shown that a significant proportion of these children are not detected by newborn hearing screening programs [2,3], school hearing screening is valuable even where universal newborn hearing screening has been implemented. Thus organizations such as the American Academy of Pediatrics [4] recommend periodic hearing screening for school-age children. In developing countries, where newborn hearing screening and preventive measures for childhood hearing loss are often unavailable, it is of utmost importance that all children be screened at school entry [5]. This is so that intervention can be carried out to minimize the adverse impacts of childhood hearing loss on well-being, development and future vocational opportunities [6-11]. In addition to early detection of hearing loss, routine school screening can also reduce the medical access barriers faced by families in rural areas and/or in developing countries [12] as they do not need to travel long distances to major cities for screening services but can gain access in their local communities.

Among all school hearing screening methods, pure-tone audiometry remains the most widely performed test worldwide. Pure-tone audiometry has served as the ‘gold standard’ for more than 50 years [13] because of its high sensitivity and specificity [14]. A commonly used passing criterion for pure-tone screening is 25 dB HL [15], which is a standard fence for normal hearing. Some screening protocols use a 20 dB HL criterion to better detect minimal hearing loss [16-19]. Nevertheless, both of these criteria are often not feasible in screening programs due to the presence of excessive ambient noise in the test setting. In usual practice, a higher cutoff value from 30 dB HL to 40 dB HL is adopted [20-23]. School hearing screening usually takes place in an enclosed, unoccupied, furnished classroom where ambient noise ranges from 30 to 64 dB A [23-30], often far exceeding the 35 dB A standard recommended by the American National Standards Institute (ANSI) [31] and the American Speech-Language-Hearing Association (ASHA) [32] for unoccupied, furnished classroom environments. Classroom noise originates from lighting and HVAC (heating, ventilation and air conditioning) systems, adjacent classrooms and external traffic noise [27,29]. Lack of acoustic treatments such as acoustic ceiling tiles, acoustically modified furniture, carpets, and double-glazed windows in most school settings further aggravates classroom background noise [33,34]. Classroom acoustics in developing countries are often particularly poor. The mean ambient noise in a public school in Brazil may be as high as 63.3 dB A [30], more than 10 dB A greater than levels reported from studies in Britain, Hong Kong and the USA. Schools in developing countries are more vulnerable to ambient noise because more basic infrastructure, such as concrete walls and bare floors [35] with the absence of a roof or walls in some cases [36], provides poor acoustic isolation. Furthermore, opening windows and doors for better ventilation allows external urban noise to easily enter [33,35,37].

Classroom ambient noise is concentrated at low frequencies (500 Hz and below) [23,29,38,39] and masks test tones, which may leave them undetected in pure-tone audiometry. This leads to high false positive findings and subsequent unnecessary diagnostic assessments. Masking, in particular of lower frequency test tones, remains a great problem for pure-tone screening in schools. Conventional TDH-39 supra-aural earphones used in pure-tone hearing screening [40] fail to eliminate low frequency (500 Hz and below) ambient noise [38,39] despite good noise attenuation ability at high frequency regions. This is because noise penetrates into the headset via cable passageways and splits between the receivers and ear cushions [41]. The low frequency region has the lowest suggested permissible noise level [42] for pure-tone hearing assessment (Table 1).

**Table 1:** Comparison of noise attenuation levels of noise-cancelling headphones and TDH-39 supra-aural earphones across frequencies

**Octave band frequency (Hz)**	Noise attenuation of noise-cancelling headphones (dB)*	Typical attenuation of TDH-39 supra-aural earphones (dB)**	**Difference in noise attenuation level (dB)**
	**A**	**B**	**(A – B)**
125	11	3	8
250	11	5	6
500	9	7	2
1000	23	15	8
2000	23	26	−3
4000	35	32	3
8000	33	24	9

* Values of Sennheiser PXC450 noise-cancelling circumaural headphones [51].

* * Values from ISO 8253–1 (1989).

With advances in technology, an active noise control (ANC) technique can now be applied to headphones and this may help mitigate the problems created by low frequency noise. The resultant noise-cancelling (NC) headphones have built-in microphones outside the headset that input external ambient noise and inside the headset that input residual noise leaking into the ear cups through cable passageways and gaps between headphones and ear cushions. Such a ‘duo microphones’ system can capture most surrounding noise and send the assembled signals to an ANC system which generates an anti-noise signal of equal amplitude but 180^o^ out-of-phase to the captured noise [43,44]. This anti-noise signal is emitted via the headset speakers and is superimposed on the primary noise signal, to cancel noise near the listener’s tympanic membrane [43-47]. In this way, much background noise is not perceived by listeners. NC headphones on average have higher noise reduction ability across nearly all frequencies than TDH-39 earphones (Table 1). Since noise is measured in a logarithmic scale, the 6 dB and 2 dB greater noise attenuation of NC headphones compared with TDH-39 earphones at 250 Hz and 500 Hz, respectively, suggests that less low frequency noise will be perceived by listeners when NC headphones are used. Noise attenuation below 500 Hz should lead to less masking effects on a 500 Hz test tone. Non-adaptive feedback ANC, an ANC design commonly found in commercial NC headphones, allows up to 20 dB noise attenuation for frequencies below 700 Hz [47].

Although NC headphone technology has been widely adopted in the audio and music industries, gaining a good reputation for effectiveness, no research has evaluated its efficacy in audiometric screening and this potential application requires investigation. In the present study it was hypothesized that the use of NC headphones would increase the specificity of school hearing screening for children. Screening with NC headphones was expected to lead to significantly lower overall referral rates and higher passing rates at 500 Hz than screening with TDH-39 earphones, for both 30 dB HL and 25 dB HL referral criteria. Passing rates at 1000, 2000 and 4000 Hz for screening were expected to be similar using both transducer types.

## Methods

### Participants

246 children, aged 6 to 8 years on the day of testing, were recruited on a voluntary basis. This age range was chosen as it matches the school entry age of most children in developing countries [48], where effective new NC headphone technology may be most needed. This age group was also included in the targeted grade levels for hearing screening advised by the American Academy of Pediatrics [4]. None of the participants reported any otological problems prior to testing. All research was performed in accordance with the Declaration of Helsinki and was approved by the Human Research Ethics Committee for Non-Clinical Faculties at the University of Hong Kong prior to participant enrollment. Written consents were obtained from each participant and their parents prior to testing. The data were collected over a period of three months within the same school year.

### Pilot study

14 normal hearing children (28 ears), nine male and five female, with a mean age of 6.7 years (S.D.: 0.64 years), were recruited from the local community. A GSI 17 audiometer was fitted with a pair of TDH-39 earphones and a pair of Sennheiser PXC450 NC headphones. This model of NC headphones was chosen as it had greater low frequency noise attenuation when compared to other models and brands available at the time of purchase. Since calibration data and specifications for the NC headphones were not provided, they were biologically calibrated with a group of normal hearing children using a calibrated GSI 17 portable screening audiometer equipped with a pair of TDH-39 earphones, using a protocol modified from Sliwa et al.’s study [19]. To avoid a practice effect, transducer type and right-left selection were randomized. The pilot study was conducted in a double-walled, sound-treated test booth. Participants were first conditioned to raise their hand when a sound was heard using a 1000 Hz tone at 60 dB HL, as this tone has good test-retest reliability [49]. When participants became familiar with the task, thresholds at four standard screening frequencies—1000 Hz, 2000 Hz, 4000 Hz and 500 Hz—were obtained sequentially. The tone intensity was varied by ±5 dB HL, starting from 30 dB HL. Thresholds were determined by obtaining two positive responses out of three trials using a modified Hughson-Westlake up-down threshold determination procedure [49]. Individual frequency specific correction factors for the NC headphones were derived for both right and left channels with reference to thresholds measured using the TDH-39 earphones (Table 2), to ensure equal output intensities for each transducer type. Mean thresholds for the pediatric listeners for TDH-39 earphones at each test frequency were obtained and were compared to the same thresholds obtained for NC headphones, with the difference between the two means used as the correction factor. These values were applied in the subsequent main study screening assessments.

**Table 2:** Correction factors for right and left channels of Sennheiser PXC450 noise-cancelling headphones with reference to TDH-39 supra-aural earphones

	**Frequency (Hz)**			
**Correction factor**	**500**	**1000**	**2000**	**4000**
**Right**	0	0	+5^*^	+10^*^
**Left**	0	+5^*^	0	0

*A positive sign indicates additional acoustic output to obtain a hearing threshold in Sennheiser PXC450 noise-cancelling headphones to be equivalent to TDH-39 supra-aural earphones.

### Main study

237 students were recruited from four mainstream primary schools in Hong Kong that agreed to take part in the study. Five participants were excluded from data analysis due to unreliable test results and/or were out of the study target age range. The final main study group was composed of 232 participants (464 ears), with 121 males and 111 females, and a mean age of 7.4 years (S.D.: 0.58 years).

All pure-tone screening audiometers (GSI 17) used in the main study were calibrated according to ANSI S3.6-1989 standards prior to use. A biological calibration check of the audiometers was also conducted by the first author before each screening session. Two calibrated GSI 17 audiometers were used to conduct hearing screening. One audiometer was fitted with NC headphones and another was equipped with TDH-39 earphones. A type 1 sound level meter (SLM) (Cesva SC-30) and Cesva Capture Studio software were used to measure and analyze the ambient noise in the test venues of the participating schools. The SLM was each day calibrated with a CB006 Class 1 acoustic calibrator with reference to IEC 60942: 2003 standards prior to measurements.

The main study was conducted in classrooms arranged by participating schools on school attendance days. All the screening test rooms were unoccupied and quiet, but not sound-treated, with all ventilation devices, windows and doors closed during testing. Visual distractions in the rooms, if any, were minimized to reduce disturbance to participants so that they could concentrate on the screening test. Ambient noise levels in the assigned classrooms were measured and analyzed using a SLM on at least three occasions, each for 5-minute intervals with sampling rate at 1s, randomly selected during the screening session.

Each participant received two hearing screenings, one using TDH-39 earphones and one with NC headphones. To avoid order effects, transducer type and right-left ear selection were randomized. Participants were first conditioned to raise their hand when they heard a sound using a 1000 Hz tone at 60 dB HL. After a few practice trials, participants were screened at 30 dB HL and 25 dB HL at the four screening frequencies. To avoid any visual cues during testing, participants were seated at right angles to the tester in both the pilot and main studies. The passing criterion was two positive responses out of three trials at each frequency at 30 dB HL and 25 dB HL, bilaterally. Failure to respond at a particular frequency at a criterion intensity was regarded as ‘did not pass’ for that frequency at that presentation level. Parents of all tested participants were given a hard copy of their child’s hearing screening report. Professional referral was provided to those who failed to respond at any frequency using a 30 dB HL criteria in either ear with conventional TDH-39 earphones.

### Data analysis

To investigate the acoustic conditions at each testing venue, overall noise levels in dB A (slow) and dB SPL, and frequency spectrum analysis in octave bands from 31.5 Hz to 16 kHz in dB SPL, were calculated by averaging the three to five samples obtained on each school visit. Descriptive methods were applied to gather demographic data of the participants. Nonparametric analysis incorporating a Pearson chi-square test or Fisher’s exact probability test was conducted to examine the overall (failed at any frequency at either ear) and frequency specific referral rates at the two screening intensities—30 dB HL and 25 dB HL—of the two transducers. Statistical tests of association between individual test results with NC headphones and TDH-39 earphones were also applied using Kappa values of agreement. Statistical significance was set at *p* = 0.05 (one-tailed).

## Results

### Ambient noise levels

Mean ambient noise levels in four primary schools are shown in Table 3. Data represents the average noise levels obtained from at least three samplings on each school visit. The noise levels were similar in the four schools and the average noise level for 90% of the test sessions (L_90_) in all schools was 43.25 dB SPL.

**Table 3:** Mean ambient noise levels in unoccupied classrooms of four primary schools

	**dB A**_**eq5min**_	**L**_**50**_	**L**_**90**_
School A	52	46	43
School B	46	42	40
School C	53	47	44
School D	49	49	46

**Figure 1:**
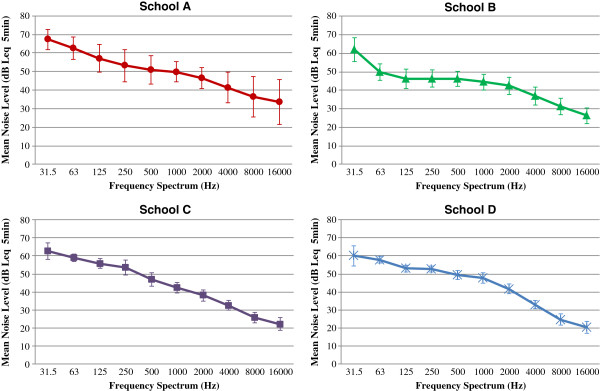
Frequency spectrum analysis of mean ambient noise in unoccupied classrooms of four primary schools, with 1 standard deviation error bars.

An overall frequency spectrum analysis of ambient noise in each classroom is given in Figure 1. Unoccupied classroom ambient noise level decreased with increasing octave band frequency. A clear predominance of low frequency noise was observed in all school settings. School B revealed a substantially reduced noise level at low frequencies compared with other schools, probably because the test venue was located in the basement of the school.

### Comparison between TDH-39 supra-aural earphones and noise-cancelling headphones

232 school children received hearing screening with both TDH-39 earphones and NC headphones. Their demographic characteristics are shown in Table 4. Table 5 shows the overall referral rates, with all frequencies included, for both transducer types decreased as age increased for screening at 30 dB HL. Nevertheless, this relationship was not statistically significant (P = 1, d.f. = 2), as revealed by Fisher’s exact test. Neither overall referral rates at 25 dB HL nor referral rates when the 500 Hz tone was excluded showed a statistically significant age effect.

**Table 4:** Age , gender and grade distribution of participants

	**Female (n = 111)**	**%**	**Male (n =121)**	**%**	**Total (n= 232)**	**%**
Age (years)						
6	19	17	35	29	54	23
7	71	64	73	60	144	62
8	21	19	13	11	34	15
Grade						
Primary 1	28	25	45	37	65	28
Primary 2	83	75	76	68	167	72

**Table 5:** **Association between age and referral rates in participants using TDH**-**39 supra**-**aural earphones and noise**-**cancelling headphones at 30 dB HL and 25 dB HL**

**Referral rates**	**No. of Refer (%)**		***χ***^**2**^	**p**-**value**
	**TDH-39**	**PXC-450**		
**500 Hz Included**				
**30 dB HL**				
6 (n= 108)	15.7% (17)	3.7% (4)	N/A^1^	1
7 (n=288)	13.5% (39)	3.5% (10)		
8 (n=68)	11.8% (8)	1.5% (1)		
**25 dB HL**				
6 (n= 108)	29.6% (32)	15.7% (17)	0.04	0.9802
7 (n=288)	26.0% (75)	12.8% (37)		
8 (n=68)	29.4% (20)	14.7% (10)		
**500 Hz Excluded**				
**30 dB HL**				
6 (n= 108)	1.9% (2)	0.9% (1)	N/A^1^	0.6786
7 (n=288)	0.3% (1)	1% (3)		
8 (n=68)	0% (0)	1.5% (1)		
**25 dB HL**				
6 (n= 108)	2.8% (3)	2.8% (3)	N/A^1^	1
7 (n=288)	3.5% (10)	4.3% (12)		
(n=68)	2.9% (2)	2.9% (2)		

N/A^1^: Fisher’s Exact Test used; d.f. = 2; α = 0.05.

Since no age effect was present, data from all age groups were combined to compare the pass/refer rates before and after excluding results of 500 Hz for both transducer types. When all frequencies were included, the referral rates for the NC headphones and the TDH-39 earphones were 3.2% and 12.9% at 30 dB HL, respectively. At 25 dB HL, referral rates of the NC headphones and the TDH-39 earphones were 13.8% and 28.2%, respectively. Results from a chi-square test or Fisher’s exact probability test, as appropriate, revealed that at both 30 dB HL and 25 dB HL criteria, referral rates before and after excluding 500 Hz results for the two transducers were statistically different—before exclusion, at 30 dB HL (P< 0.05, d.f. = 1) and at 25 dB HL (*χ*^2^ = 28.76, P < 0.05, d.f. = 1); after excluding 500 Hz, at 30 dB HL (P< 0.05, d.f. = 1) and 25 dB HL (P< 0.05, d.f. = 1) (Table 6). Kappa observed agreement (OA) of the 500 Hz inclusion group (at 30 dB HL: OA = 0.864; at 25 dB HL: OA = 0.735) was smaller than that of the 500 Hz exclusion group (at 30 dB HL: OA = 0.991; at 25 dB HL: OA = 0.946). This indicates that TDH-39 earphones and NC headphones differed in screening outcome when 500 Hz results were included. In the 500 Hz exclusion group, the discrepancies between Fisher’s exact test and OA results can be attributed to the small cell size, 5 or below, when the two transducers obtained opposite results, i.e., pass for one and fail for the other, as majority of participants passed with both the TDH-39 earphones and the NC headphones. This would affect Fisher’s exact test analysis and therefore, OA results should be given greater weight.

**Table 6:** **Comparison of the overall pass and referral rates between TDH**-**39 supra**-**aural earphones and Sennheiser PXC450 noise**-**cancelling headphones before and after excluding screening results at 500 Hz at 30 dB HL and 25 dB HL**

**No. of ears (n**=**464)**	**No. of pass (%)**		**No. of refer (%)**		**Observed agreement**	**Kappa**	***χ***^**2**^	**p**-**value**	**Odds ratio**
	**TDH-39**	**PXC-450**	**TDH-39**	**PXC-450**					
**Before excluding 500 Hz**									
**30 dB HL**	404 (87.1%)	449 (96.8%)	60 (12.9%)	15 (3.2%)	0.864	0.1142	N/A^1^	0.0072	5.88
**25 dB HL**	333 (71.8%)	400 (86.2%)	131 (28.2%)	64 (13.8%)	0.735	0.2257	28.76	< 0.0001	4.13
**After excluding 500 Hz**									
**30 dB HL**	461 (99.4%)	459 (98.9%)	3 (0.6%)	5 (1.1%)	0.991	0.4959	N/A^1^	0.0003	305.33
**25 dB HL**	449 (96.8%)	446 (96.1%)	15 (3.2%)	18 (3.9%)	0.946	0.2147	N/A^1^	0.0017	11.30

N/A^1^: Fisher’s Exact Test used; d.f. =1; α = 0.05.

In order to investigate whether NC headphones and TDH-39 earphones screen similarly, referral rates at the individual frequencies of 500 Hz, 1000 Hz, 2000 Hz and 4000 Hz were also compared using chi-square or Fisher’s exact test. Results in Table 7 show that the two transducers screened similarly at 1000 Hz (P > 0.05, d.f. = 1) and 2000 Hz (P > 0.05, d.f. = 1) at 25 dB HL. No statistical difference was found for 4000 Hz at both 30 dB HL (P > 0.05, d.f. = 1) and 25 dB HL (P > 0.05, d.f. = 1) criteria. However, statistical significant differences were observed for 500 Hz at 30 dB HL (P < 0.05, d.f. = 1) and at 25 dB HL (*χ*^2^ = 34.86, P < 0.05, d.f. = 1), 1000 Hz (P < 0.05, d.f. = 1), and 2000 Hz (P < 0.05, d.f. =1) at 30 dB HL. When OA was considered, it showed that the two transducer types had almost perfect agreement, i.e., they screened similarly at all frequencies (e.g., 1000 Hz: OA = 0.996) except at 500 Hz (at 30 dB HL: OA = 0.873; at 25 dB HL: OA = 0.750). Larger discrepancies between Fisher’s exact test and OA at 1000 Hz and 2000 Hz were again influenced by the small cell size, 5 or below, when the two transducers obtained opposite results, as previously mentioned.

**Table 7:** Comparison of the pass and referral rates at individual frequencies for TDH-39 supra-aural earphones and Sennheiser PXC450 noise-cancelling headphones at 30 dB HL and 25 dB HL

**No. of ears (n=464)**	**No. of pass (%)**		**No. of refer (%)**		**Observed agreement**	**Kappa**	***χ***^**2**^	**p**-**value**	**Odds ratio**
	**TDH-39**	**PXC-450**	**TDH-39**	**PXC-450**					
**500 Hz**									
30 dB HL	406 (87.5%)	453 (97.6%)	58 (12.5%)	11 (2.4%)	0.873	0.109	N/A^1^	0.0066	6.29
25 dB HL	335 (72.2%)	413 (89%)	129 (27.8%)	51 (11%)	0.750	0.245	34.86	<0.0001	5.49
**1000 Hz**									
30 dB HL	463 (99.8%)	462 (99.6%)	1 (0.2%)	2 (0.4%)	0.996	0.004	N/A^1^	0.0043	∞
25 dB HL	450 (97%)	451 (97.2%)	14 (3%)	13 (2.8%)	0.950	0.123	N/A^1^	0.0546	6.65
**2000 Hz**									
30 dB HL	461 (99.4%)	462 (99.6%)	3 (0.6%)	2 (0.4%)	0.998	0.80	N/A^1^	0.0000	∞
25 dB HL	461 (99.4%)	461 (99.4%)	3 (0.6%)	3 (0.6%)	0.996	0.75	N/A^1^	6.0452	∞
**4000 Hz**									
30 dB HL	464 (100%)	462 (99.6%)	0 (0%)	2 (0.4%)	0.994	1	N/A^1^	1	N/A
25 dB HL	464 (100%)	459 (98.9%)	0 (0%)	5 (1.1%)	0.987	1	N/A^1^	1	N/A

N/A^1^: Fisher’s Exact Test used; d.f. = 1; α = 0.05.

## Discussion

### Effect of ambient noise on school screening

Unoccupied classrooms with furniture only are quieter than occupied classrooms, and are usually chosen for school hearing screening. Nevertheless, such so-called quiet venues usually fail to meet the 35 dB A upper limit recommended by ANSI [31] and ASHA [32] for unoccupied furnished classroom noise level. In the present study, mean overall ambient noise level and L_90_ measured in four urban mainstream primary schools ranged from 46 to 52 dB LA_eq 5 min_ and 40 – 46 dB SPL, respectively. This noise level is approximately 10 dB A above the published guidelines, and these findings were comparable to previous studies in other schools [26-29,33,37]. Spectrum analysis revealed that classroom ambient noise was predominately at low frequencies (Figure 1). The noise level at 250 Hz and 500 Hz was of most concern as this range exerts the greatest masking effect on 500 Hz test tones in screening. In this study, the average 250 Hz and 500 Hz background noise levels in the four schools were 51.25 dB LZeq _5min_ and 48.5 dB LZeq _5min_, respectively. Such intensity levels are much higher than the intensity of the 25dB HL screening stimuli, leaving 500 Hz difficult to detect. This may well account for the highest referral rate—27.8%—associated with 500 Hz screening frequency when 25 dB HL was the passing criterion and TDH-39 earphones were used.

### Utility of noise-cancelling headphones in school hearing screening

In order to examine the effectiveness of NC headphones in counteracting the masking effect of ambient noise during hearing screening, the overall referral rates of pure-tone screening both including and excluding 500 Hz results were compared. When a 30 dB HL passing criterion was applied, the overall referral rates of TDH-39 earphones including and excluding 500 Hz results were 12.9% and 0.6%, respectively. A large reduction in referral rate of 12.3% was revealed. However, the difference in referral rates for the NC headphones with and without 500 Hz was much smaller than that of the TDH-39 earphones, with only a 2.1% difference (from 3.2% to 1.1%). A much larger difference for the TDH-39 earphones than that of the NC headphones suggested that the former was much more susceptible to ambient noise effects. When a more stringent pass/refer criterion—25 dB HL—was adopted, it was expected that the referral rate difference before and after exclusion of 500 Hz results would widen in both transducer types as the 500 Hz tone became harder to detect as the signal to ambient noise ratio was reduced. The degree of difference between 500 Hz results included and excluded was much greater in TDH-39 earphones (25%; from 28.2% to 3.2%) than NC headphones (9.9%; from 13.8% to 3.9%). This further confirmed that the TDH-39 earphones were more affected by ambient noise, which led to higher fail counts. TDH-39 earphones are more vulnerable to background noise than NC headphones because they attenuate noise by passive shielding—maintained by contact between the MX-4I/AR rubber earphone cushion and the pinna through pressure exerted by the earphone headband. In contrast, NC headphones directly eliminate low frequency ambient noise by generating an equal amplitude but completely out of phase signal to cancel the primary noise signal. This approach allows NC headphones to effectively eliminate steady noise types as the anti-phase signal is locked to the noise source by real-time noise capture and analysis via the ‘duo microphones’ and ANC system.

When 500 Hz results were included, a smaller OA between the two transducers was found for the 25 dB HL criterion (OA = 0.735) when compared to 30 dB HL (OA = 0.864). Similar findings were observed when 500 Hz results were analyzed alone—smaller OA with 25 dB HL criterion (OA = 0.75) than 30 dB HL screening level (OA = 0.873). This indicates that differences in referral rates for the two transducers in this study were greater with lower screening intensity. Evidence that NC headphones screened more effectively at the more stringent passing criterion than TDH-39 earphones when a low frequency pure-tone was included in the protocol supports the use of NC headphones if a screening program includes a 500 Hz test tone at 25 dB HL. The capability of NC headphones to operate at lower screening intensities gives higher screening sensitivity with test specificity maintained. ASHA modified its screening 1997 guidelines by excluding a 500 Hz test tone, which was previously included in its 1990 guidelines, because of ambient noise considerations [15,50]. This has also been routinely done in many school screening programs outside North America due to the high false positive findings generated as a consequence of the masking effect of ambient noise [5,51-53]. However, this practice is not preferred as it may leave otitis media or other conductive loss undetected since low frequency acuity is a good indicator of middle ear integrity [54]. Otitis media is a common cause of hearing loss in young children [55], particularly in developing countries. A prevalence rate of 9.4% to 25.5% has been noted in a range of developing nations [22,56-62]. With the use of NC headphones, it may be feasible to include a 500 Hz test tone in school settings even with the presence of low frequency background noise.

Identification of mild hearing loss may also be more practicable when NC headphones are used as they allow screening protocols to adopt a lower screening intensity level. Research suggests that mild hearing loss in children may lead to substantial difficulties in auditory perception—including speech discrimination, recognition and hearing in noise difficulties [63,64], as well as speech and language disorders [8,65]. Early detection of mild hearing loss allows implementation of remedial strategies to facilitate a child’s learning. Even in developing countries where amplification systems are unavailable, measures as simple as preferential seating in the classroom may benefit identified children a great deal. Results in this study favor the possible use of NC headphones at the more stringent criterion—25 dB HL. Future studies could explore the possibility of lowering the intensity to 20 dB HL as this level can further increase screening sensitivity and more effectively identify slight to mild hearing loss.

A shortcoming of NC headphones is that there is a lack of calibration specifications, which makes psychoacoustic calibration with a group of normal hearing individuals necessary prior to audiometric use. Specific calibration information that readily enabled NC headphone output to be compared to that of TDH-39 earphones, at audiometric test frequencies, would be valuable. Also, provision of frequency response curves for NC headphones and noise-attenuation information at a wide range of frequencies (e.g., octave band frequencies from 31.5 Hz to 16000 Hz) would make comparison of noise reduction capabilities amongst different NC headphones more convenient. If specific calibration is not available then improved biological calibration is important. The present study developed individual frequency specific correction factors for the NC headphones based on a small sample only of paediatric listeners with normal hearing.

### Noise attenuation of noise-excluding headphones, TDH-39 supra-aural earphones and noise-cancelling circumaural headphones

Some hearing screening protocols have used noise-excluding headphones, i.e., TDH-39 earphones mounted inside circumaural audiocups (TDH-39/A headphones) instead of TDH-39 earphones alone, for extra attenuation [18,23,38,55,60]. TDH-39/A headphones provide greater noise attenuation as the audiocups thoroughly enclose the entire pinna with a soft plastic cushion [66,67] to reduce the chances of pure-tone leakage and noise entry. The principle used to achieve noise attenuation with audiocups is, however, similar to that of TDH-39 earphones and is based on the assumption that the cushion completely seals the ear while in reality, due to anatomical differences of the head and pinna among listeners, gaps can hardly be avoided. Therefore, it is expected that TDH-39/A headphone noise-attenuation ability will be poorer than that of noise-cancelling headphones. An early study comparing the attenuation characteristics of noise-excluding headphones (‘Otocups’ Mark III) and TDH-39 earphones showed that the former had approximately 10 dB greater mean noise attenuation than the TDH-39 earphones across frequencies [67]. The measured mean attenuation values at 500 Hz for TDH-39 earphones enclosed in ‘Otocups’ shells and TDH-39 earphones alone were 15 dB and 7 dB, respectively. Such attenuation data pointed to a large attenuation gain with noise-excluding circumaural headphones compared with conventional headphones at low frequencies. However, when the standard deviations at 500 Hz for this early study are taken into account (7.5 dB for ‘Otocups’ and 9.2 dB for TDH-39 earphones) there was not a great difference in noise attenuation between the two transducers. The large intrasubject variation observed in mean attenuation values with audiocups and TDH-39 earphones might be due to headphone positioning effects. In a recent study, it was pointed out that a headset that physically excludes noise does not automatically guarantee accurate hearing threshold measurement, due to calibration issues. Calibration of TDH-style earphones using a 6cc coupler is based on the assumption that the receiver and its ear cushion is in close contact with the pinna. However, it is hard to mount TDH-style earphones that are inside audiocups in an optimal position, so that when placed on listeners the earphones seal the ears well but loosely cover the pinna [41]. Due to this issue, TDH-39/A headphones and TDH-39 earphones may in practice show similar noise attenuation capabilities. However, further research that explicitly compares the noise attenuation performance of TDH-39 earphones, TDH-39/A earphones and NC headphones in a school hearing screening environment is needed before a truly informed choice of optimal school hearing screening headphones can be made. When comfort factors are considered, TDH-39/A headphones are less optimal than NC headphones as the latter (315 g, battery included) are approximately half the weight of the 620 g TDH-39/A headphones, due to the absence of the bulky noise-excluding shells. Also, NC headphones do not need to be positioned as tightly as noise-excluding headphones on a child’s head, and thus may cause less discomfort to young children. This is because NC headphones do not rely on a tight seal between the cushion and ear to exclude noise but rather create a quiet listening environment around the listener ear by phase cancellation.

### Potential value of noise-cancelling headphones in developing countries

Environmental test conditions as well as tester and equipment availability are important factors for effective implementation of hearing screening programs in developing countries [21]. School hearing screening usually takes place in far from ideal conditions which are affected by a considerable amount of ambient noise, predominately at low frequencies. Since the environment is usually hard to modify, selection of appropriate screening technology is a practical way to tackle the noise problem. NC headphones have potential to replace TDH-39 earphones in school screening because they actively eliminate ambient noise. Alternatively, one could choose insert earphones to replace conventional headphones for school screening as they have better noise attenuation [68]. Nonetheless, the foam tips used in insert earphones are disposable and this recurrent expenditure is expensive in both developing and developed economies. In addition, large concentrations of cerumen are common in school children, particularly in developing countries where rates for impacted cerumen can be as high as 52.6% [69]. The small diameter sound bore in insert earphones is prone to blockage by even minor amounts of cerumen, leading to false positive screening outcomes. For these reasons insert earphones are not advised for use in school hearing screening programs [70].

For selection of screening tools, cost is an important consideration particularly for health workers in developing countries. Results from a Google search showed that the retail price of new set of TDH-39/A headphones offered by medical equipment vendors is at least $US 355 (shipping excluded). The price of TDH-39 earphones was not determined as they are usually provided with purchase of a screening audiometer. The NC headphones (Sennheiser PXC450) used in this study had the highest specification among all available models and brands in the market at the time of purchase and cost $US 410. There were other brands of NC headphones with lesser noise attenuation specifications which were much more affordable. Although the current cost of NC headphones is higher than that of TDH-39/A headphones, the price of NC headphones is expected to decrease in future due to keen competition in the commercial market and the wide application of noise-cancelling technology.

Although a standard AAA battery is needed to drive NC headphones, this type of battery can be easily obtained in most developing countries. NC headphones require little power to function and frequent battery replacement is not necessary. In this study, only two alkaline cells were used to screen more than 200 students. The use of rechargeable batteries to replace alkaline cells could reduce the ongoing cost of battery replacement.

## Conclusions

NC headphones had significantly lower overall referral rates (with 500 Hz results included) than the TDH-39 earphones at both 30 dB HL and 25 dB HL criteria. Similar results were found for referral rates at exclusively 500 Hz. When mid and high frequencies (1000 Hz to 4000 Hz) were considered, both NC headphones and TDH-39 earphones had comparable referral rates. This suggests that NC headphones may be a promising alternative to TDH-39 earphones for hearing screening in schools due to their higher resistance to low frequency ambient noise and light weight. With NC headphones, audiologists or screening professionals may not need to adopt loose screening criteria because of the unfavorable noise screening conditions often found in school settings. Screening at lower intensity levels becomes possible with NC headphones without compromising screening specificity. Future large scale studies that compare the noise attenuation of NC headphones and TDH-39/A equipment, as well research on the implications of a further reduced pass /refer criteria of 20 dB HL, will provide more information on appropriate headphone selection for optimal school hearing screening test accuracy.

## Competing interests

The authors declare that they have no competing interests.

## Authors’ contributions

AL undertook the data collection, statistical analysis and drafted the original manuscript. BM developed the initial study design and supported data analysis. Both authors read and approved the final manuscript.

## Pre-publication history

The pre-publication history for this paper can be accessed here:

http://www.biomedcentral.com/1472-6815/13/6/prepub
